# Combined Immune Defect in B-Cell Lymphoproliferative Disorders Is Associated with Severe Infection and Cancer Progression

**DOI:** 10.3390/biomedicines10082020

**Published:** 2022-08-19

**Authors:** Juliana Ochoa-Grullón, Kissy Guevara-Hoyer, Cristina Pérez López, Rebeca Pérez de Diego, Ascensión Peña Cortijo, Marta Polo, Marta Mateo Morales, Eduardo Anguita Mandley, Carlos Jiménez García, Estefanía Bolaños, Belén Íñigo, Fiorella Medina, Antonia Rodríguez de la Peña, Carmen Izquierdo Delgado, Eduardo de la Fuente Muñoz, Elsa Mayol, Miguel Fernández-Arquero, Ataúlfo González-Fernández, Celina Benavente Cuesta, Silvia Sánchez-Ramón

**Affiliations:** 1 Department of Clinical Immunology, Institute of Laboratory Medicine and IdISSC, Hospital Clínico San Carlos, Calle Profesor Martín Lagos SN, 28040 Madrid, Spain; 2 Department of Hematology, Institute of Laboratory Medicine, Hospital Clínico San Carlos, 28040 Madrid, Spain; 3 Laboratory of Immunogenetics of Human Diseases, IdiPAZ Institute for Health Research, 28046 Madrid, Spain

**Keywords:** B cell chronic lymphoproliferative disorders, severe infections, secondary immunodeficiency, predominantly antibody defect, combine immune defect, cancer progression

## Abstract

B cell chronic lymphoproliferative diseases (B-CLPD) are associated with secondary antibody deficiency and other innate and adaptive immune defects, whose impact on infectious risk has not been systematically addressed. We performed an immunological analysis of a cohort of 83 B-CLPD patients with recurrent and/or severe infections to ascertain the clinical relevance of the immune deficiency expression. B-cell defects were present in all patients. Patients with combined immune defect had a 3.69-fold higher risk for severe infection (*p* = 0.001) than those with predominantly antibody defect. Interestingly, by Kaplan–Meier analysis, combined immune defect showed an earlier progression of cancer with a hazard ratio of 3.21, than predominantly antibody defect (*p* = 0.005). When B-CLPD were classified in low-degree, high-degree, and plasma cell dyscrasias, risk of severe disease and cancer progression significantly diverged in combined immune defect, compared with predominantly antibody defect (*p* = 0.001). Remarkably, an underlying primary immunodeficiency (PID) was suspected in 12 patients (14%), due to prior history of infections, autoimmune and granulomatous conditions, atypical or variegated course and compatible biological data. This first proposed SID classification might have relevant clinical implications, in terms of predicting severe infections and cancer progression, and might be applied to different B-CLPD entities.

## 1. Introduction 

A major challenge in B cell lymphoproliferative diseases (B-CLPD) is to elucidate the underlying mechanisms of the heterogeneous biological and clinical behaviour of the disease [1], not only because of molecular alterations on B cells but also from their interactions with the tumour microenvironment [2]. 

A main cause of morbidity and mortality in patients with B-CLPD is due to infectious complications. Over the last 40 years, several milestones have propelled improvement in the diagnosis and management of secondary immunodeficiency (SID) to B-CLPD: (i) the pivotal studies on prophylaxis with immunoglobulin replacement therapy (IgRT) in the pre-rituximab era [3,4,5,6,7,8]; (ii) the introduction of the anti-pneumococcal conjugate vaccine followed by 23-polysaccharide vaccine in the CDC vaccination schedule recommendations [9]; (iii) the specific antibody (Ab) response for the indication of IgRT in patients with severe or recurrent infections after antibiotic failure [10,11]; and (iv) the utility of pure polysaccharide *Salmonella typhi* vaccine (TV) as a complementary tool to evaluate primary vs. secondary responses in SID patients [12,13,14]. 

SID occurs concomitantly with tumor growth and immune escape mechanisms, impairing normal B cell maturation, and secondarily as a consequence of tumor therapies. However, no biomarkers of SID are included in current prognostic scores for B-CLPD, nor consensus on the evaluation of immune status unless infections show up [15,16,17,18,19]. Thus, it is reasonable to raise the question of whether immunodeficiency might predict not only infectious risk but also B cell cancer progression or recurrence [20]. 

From an immunological point of view, B-CLPD course and its treatments induce a B cell defect with predominantly humoral immune deficiency. Nevertheless, other immune cell types and proteins, such as CD4^+^ and CD8^+^ T-cells, myeloid suppressor cells, dendritic cells (DCs), natural killer (NK) cells, and the complement system can be profoundly altered as to render the patient susceptible to increased risk of viral and opportunistic infections [21,22]. The impact of B-CLPD-associated immune defects beyond B cell defects on the clinical outcomes has not been fully addressed. The purpose of this investigation, in a close collaborative effort with haematologists, was to ascertain whether immunological stratification of SID-B-CLPD patients, as performed in primary immunodeficiency (PID) based on the main immune branch affected (B/T/NK cells/complement/phagocytes) may be clinically relevant. Here we provide a comprehensive clinical and immunological overview of a real-life cohort of SID-B-CLPD patients to build on infectious risk stratification, but also on second primary neoplasia, autoimmunity, and gastrointestinal involvement. Management of SID patients include IgRT and prophylactic antibiotics [23,24,25]. Trained immunity-based vaccines (TIbV) might be a promising adjuvant strategy to reduce infections in SID patients [26,27,28].

In other order of things, a yet unknown proportion of SID patients may be a B-CLPD onset of the PID. The implementation of an immunological work-up at diagnosis of B-CLPD represents an opportunity for unveiling PID when suspected [20]. Fine-tuned evaluation of SID patients might encompass innate and adaptive immunity defects and would also be of great value to facilitate further clinical research and comparative studies.

## 2. Methods

### 2.1. Patients and Study Design 

This is a single centre retrospective observational cohort study conducted at the Hospital Clínico San Carlos of Madrid from 2015 to 2020. All B-CLPD patients were consecutively referred to the Clinical Immunology Department from the Haematology Department after presenting with recurrent or severe infections and/or after the finding of hypogammaglobulinemia. None of the patients were under active treatment at the time of the immunological assessment. The diagnosis of B-CLPD was performed based on the WHO Classification of Tumours of Haematopoietic and Lymphoid Tissue [29]. The diagnosis of SID was performed based on recent clinical guidelines [23,30,31]: recurrent or severe infections, reductions of serum IgG, IgA, and/or IgM by two or more standard deviations from the normal mean, and failure to mount Ab response to polysaccharide and/or protein antigens. Definition on infection data (recurrent, severe and persistent infections, etc.) were adopted based on consensus [32]. Infectious data were defined as: (i) recurrent upper respiratory tract infection (URTI): as greater than or equal to three episodes of rhinitis, sinusitis, otitis, pharyngitis, or tonsillitis; (ii) recurrent lower respiratory tract infection (LRTI) as greater than or equal to one episode of acute bronchitis, pneumonia or community-acquired pneumonia, acute exacerbation of chronic obstructive pulmonary disease (COPD) or bronchiectasis. Severe infection comprised sepsis, meningitis, osteomyelitis and complicated pneumonia. Follow-up time was counted as the time between patient referral to the Immunology Department and the date of last contact. Data were collected conforming medical records from outpatient clinics. Approval for the study was obtained from the hospital institutional research Ethics Committee (19/219-E). For those who had died, the cause of death was determined by review of the clinical history and/or by contacting the attending physician. 

### 2.2. Laboratory Assessment 

Baseline assessment included differential white blood cell count, serum immunoglobulins (IgG, IgA, and IgM), IgG subclasses (IgG1, IgG2), polysaccharide and protein IgG specific Ab responses, C3 and C4 concentrations, serum free light chains (sFLC) and lymphocytes subpopulations (CD4^+^ and CD8^+^ T-lymphocytes, CD4/CD8 ratio, CD19^+^, CD56^+^ cells). In order to evaluate specific Ab responses, patients were vaccinated with 23-valent pneumococcal polysaccharide (PPV) vaccine, Typhim Vi polysaccharide (TV) vaccine and tetanus-toxoid (TT) vaccine. Blood samples were obtained on day 0 prior to vaccination and on day 30 after vaccination. Serum was collected and stored at −40 °C until simultaneous performance of specific Ab tests. For almost all subjects, this was before treatment with IgRT was started. Specific Ab concentrations were reported for PPV IgG as mg/dL (range, 0.33–27), TV IgG as U/mL (range, 7.4–600.0) and TT IgG as U/mL (0.1–7.0). Responders were defined as individuals obtaining a fold increase (FI) ≥ 3 for PPV and TT and ≥5 for TV according to published data in PID and SID, respectively [14]. Protective Ab concentrations were defined as 4.4 mg/dL for PPV IgG and 0.15 IU/mL for TT IgG [13]. No protective Ab concentrations have been defined for TV vaccination at present. Further assessment such as serological tests and peripheral blood PCR for EBV and CMV were performed. Visits were documented every 6 months, and the observation period was at least 1.5 years per patient. 

#### 2.2.1. Immunoglobulins’ and Complement Analysis

Total immunoglobulins, IgG subclasses and C3 and C4 complement factors were measured by turbidimetry using commercial kits on an Optilite analyser (The Binding Site Group Ltd., Birmingham, UK). Reference values for IgG were: 600–1600 mg/dL, IgA: 70–400 mg/dL, IgM: 40–200 mg/dL, IgG1: 382–930 mg/dL, IgG2: 240–700 mg/dL, C3: 70–140 mg/dL and C4: 15–30 mg/dL.

#### 2.2.2. Measurement of B, T and NK Lymphocytes

EDTA anticoagulated peripheral blood samples were tested by lyse-no-wash method on a routinely calibrated FACSCanto II flow cytometer (Becton-Dickinson, San Jose, CA, USA) using a certified BD Multitest TBNK kit, and data analyzed using BD FACS software version 8.0 (Becton-Dickinson, San Jose, CA, USA) By default, the number of events acquired for each sample in our laboratory was greater than 10.000; T, B and NK cells analyses were performed simultaneously; and the median of events was 14,413 (range, 11,000–16,908). The reports contained CD4^+^ and CD8^+^ T-lymphocytes, B and NK cell percentages and absolute counts expressed as % of total lymphocytes and absolute counts (cells/mm^3^). Reference values for <65 years old were: CD4^+^ T-lymphocytes: 51–66%, 464–1721 cells/mm^3^; CD8^+^ T-lymphocytes: 28–36%, 178–853 cells/mm^3^; CD19 B lymphocytes: 7–13%, 92–515 cells/mm^3^; NK cells CD3^−^CD56^+^CD16^+^: 8–19%, 82–594 cells/mm^3^. The reference range for lymphocyte subsets in patients ≥65 years old were based on a referenced Spanish healthy population of the same age [33].

#### 2.2.3. Measurement of Differential White Blood Cell Count 

Peripheral blood cell counts were measured using the Beckman Coulter DxH 800 AU Chemistry Analyzer (Beckman Coulter Inc., Brea, CA, USA). The normal ranges were considered as follows: neutrophils 40–80%, 2.0–7.0 × 10^3^/µL; lymphocytes 20–40%, 1.0–3.0 × 10^3^/µL; and monocytes 2.0–10.0%, 0.20–1.0 × 10^3^/µL. 

### 2.3. Statistical Analysis

Descriptive data and continuous variables were recorded as mean ± standard deviation (SD) or median values (range, max–min), according to the normal or non-normal distribution of the data. Categorical variables were described as counts and percentages of subjects. Chi-squared test or Fisher’s exact test for categorical variables, were used as appropriate. Data were analyzed using Excel spreadsheet (Microsoft, Inc., Redmond, WA, USA). The Kaplan–Meier model was used to determine the probabilities of survival and progression-free interval after diagnosis of B-CLPD. The Cox proportional hazard was used to determine the difference ratio of factors that might be associated with increased risk of death and progression, adjusted for age, clinical stages and oncology treatment. The main variables we used for the multivariate analysis were serum Ig levels, Ab specific response, and C3, C4, T, NK and B cells. Statistics were analyzed with SPSS (Chicago, IL, USA) and GraphPad Prism software (GraphPad Software, La Jolla, CA, USA, version 8). Differences were considered statistically significant at *p* < 0.05.

## 3. Results

### Overall Overview 

**Figure 1:**
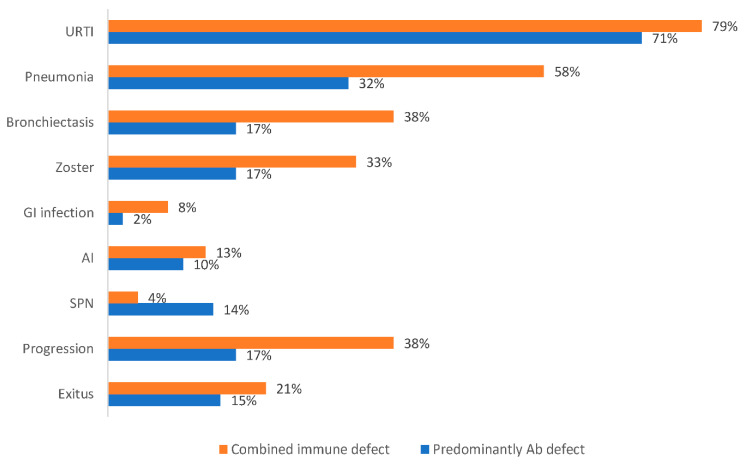
Proportion of clinical manifestations according to the immune defect phenotype.

From 2015 to 2020, 83 B-CLPD adult patients referred to the Clinical Immunology Department due to SID suspicion were evaluated. The age range was 27–91 years (mean, 67.59 ± 14.50 years), 48 (58%) females, followed for a period of 1–5 years. The mean age at the time of diagnosis was 55.87 ± 15.09 years. No differences were observed among immunologic parameters according to sex or age distribution. All patients suffered from recurrent infections. Patients were classified according to the underlying haematologic disease as: non-Hodgkin lymphoma (NHL) (*n* = 41; 49%), chronic lymphocytic leukaemia (CLL) (*n* = 18; 22%), monoclonal gammopathy of undetermined significance (MGUS) (*n* = 12; 14%), multiple myeloma (MM) (*n* = 6; 7%), Hodgkin lymphoma (HL) (*n* = 3; 4%), Waldenström disease (WD) (*n* = 2; 2%) and lymphoblastic acute leukaemia (LAL) (*n* = 1; 1%). Supplementary Table S1 shows B-cell lymphoproliferative neoplasia classification according to hematological disease. Most patients (65; 78%) had received chemotherapy or immunotherapy, while 18 were on a watch and wait strategy (WAW): MGUS (*n* = 12), CLL (*n* = 5) and FL (*n* = 1). At least half of the treated patients (*n* = 33) had received more than one cycle. Sixty-three percent (*n* = 41) received rituximab as first-line or maintenance therapy. Chemotherapies regimens protocols corresponded to: fludarabine, cyclophosphamide and rituximab (FCR); bendamustine and rituximab (BR); cyclophosphamide, doxorubicin, vincristine, and prednisone (CHOP); adriamycin, bleomycin, vinblastine, dacarbazine (ABVD) and melphalan for MM patients. In one patient with relapsing CLL, immunotherapy with ibrutinib was initiated. The mean time interval between diagnosis of malignancy and progression was 6.2 ± 4.5 (range, 1 to 17) years and the time since last chemotherapy and immunological assessment was of 4.7 ± 5.0 years (range, 0 to 17). To date, 69 patients remained alive and 14 had died. Mortality was higher among males (71.4%) than females (28.5%) (*p* = 0.016). The most frequent causes of death were cancer progression (10 of 14) and infectious complications (4 of 14). Overall, severe infection occurred in 27% (*n* = 22), 24% (*n* = 20) had varicella-zoster (VZV) infection, 11% (*n* = 9) second primary neoplasia, 10% (*n* = 8) autoimmune disease, 8% (*n* = 7) malabsorption syndrome, and 5% (*n* = 4) viral hepatitis (Figure 1).

## 4. Immunological Assessment 

### 4.1. Serum Immunoglobulins

At the time of immunological evaluation, median serum IgG was 535 mg/dL, IgA 74 mg/dL, and IgM 54 mg/dL. The median time from B-CLPD diagnosis until immunological evaluation was 7 ± 4 years. By this time, 51% (*n* = 32) had IgG levels below 400 mg/dL. For serum IgM, 65% (*n* = 41) presented values below 25 mg/dl and for serum IgA, 51% (*n* = 32) had values below 10 mg/dL. IgG levels correlated with serum IgA (r = 0.385; *p* < 0.002) and IgM (r = 0.457; *p* < 0.0001) levels. Immunological characteristics of the main study subjects are given in Table 1. No significant difference was observed in serum IgG between males and females for age distribution (*p* = 0.685). Forty-three (68%) (excluding plasma cell dyscrasias) patients had both IgG1 and IgG2 subclasses affected. Isolated low IgG1 was found in nine patients and IgG2 in three patients.

### 4.2. Specific Antibody Responses to Immunisation 

Poor responses to T-dependent (tetanus and diphtheria toxoid) and T-independent (pneumococcus and *S. Typhi*) vaccines were found in 78 individuals. Forty-seven patients (60%) had high baseline TT and/or PPV titres. Five (7%) patients presented an adequate polysaccharide antibody response to TV (Supplementary Table S2). From a clinical point of view, specific Ab responses to TV better correlated with protection against pneumonia (*p* = 0.35) and severe infection (*p* = 0.06).

### 4.3. Lymphocyte Subpopulations 

T cell abnormalities were common: CD4^+^T-lymphocytes were normal in 31% (*n* = 26) of our B-CLPD patients; between 500 and 700 cells/mm^3^ in 24% (*n* = 20); between 200 and 499 cells/mm^3^ in 34% (*n* = 28); and below 200 cells/mm^3^ in 11% (*n* = 9). CD8+ lymphocytopenia (<178 cell/mm^3^) was observed in seven patients (8%). Twenty-seven patients (32%) presented low B cells counts (<92 cell/mm^3^). Low numbers of circulating NK cells (<82 cell/mm^3^) were detected in eight patients (10%).

### 4.4. Neutrophils

We observed that 84% (*n* = 70) of our patients had neutrophils within normal range, 8% (*n* = 7) had levels between 1000 and 1500 cells/mm^3^ (mild neutropenia); 5% (*n* = 4) had levels between 500 and 999 cells/mm^3^ (moderate neutropenia); and 2% (*n* = 2) had below 500 cells/mm^3^ (severe neutropenia). Five out of six patients with moderate to severe neutropenia associated severe infection (odds ratio [OR], 13.33; 95% CI, 1.40 to 126.98 (*p* < 0.024)). 

### 4.5. Serum Complement Factors 

Decreased C4 complement factor levels (8.5 ± 3.7, range 5.0–12.0 mg/dL) were found in nine patients (11%), three of them in the setting of autoimmune disease (Sjögren syndrome, autoimmune haemolytic anaemia (AIHA), and immune thrombocytopenia (ITP)). Four patients out of nine (44.4%) with decreased C4 concentrations (7.5 ± 4.0; range, 3.2–12.0 mg/dL) passed away due to disease progression and infectious complications.

## 5. Immunological Stratification of SID 

We sought to determine whether stratifying the immune defect at B-CLPD diagnosis influenced the risk of infections and other associated conditions. All B-CLPD patients in our cohort showed humoral immune defect, either hypogammaglobulinemia or proven specific antibody failure: 71% without other apparent immune defect, namely, “predominantly antibody defect”, by analogy with PID category; while 24 (29%) of the patients associated diverse immune defects (C^−^, NK^−^ Neu^−^, or T^−^) involving cellular and innate immunity, namely, “combined immune defect”. Seven patients (8%) showed an associated decrease in C4 complement factor, six (7%) had T lymphocytopenia, five (6%) had NK defect, and the remaining five patients had three or more combined immune defects (B^−^C^−^NK^−^, B^−^C^−^NK^−^Neu, B^−^T^−^NK^−^, B^−^T^−^Neu^−^). 

Interestingly, patients with combined immune defect presented an OR of 3.69-fold higher risk for severe infection (95% CI, 1.30 to 10.40) (*p* = 0.001) and 5.30-fold higher risk of COVID-19 (95% CI, 1.67 to 17.0) (*p* = 0.004) than predominantly antibody defect.

**Figure 2:**
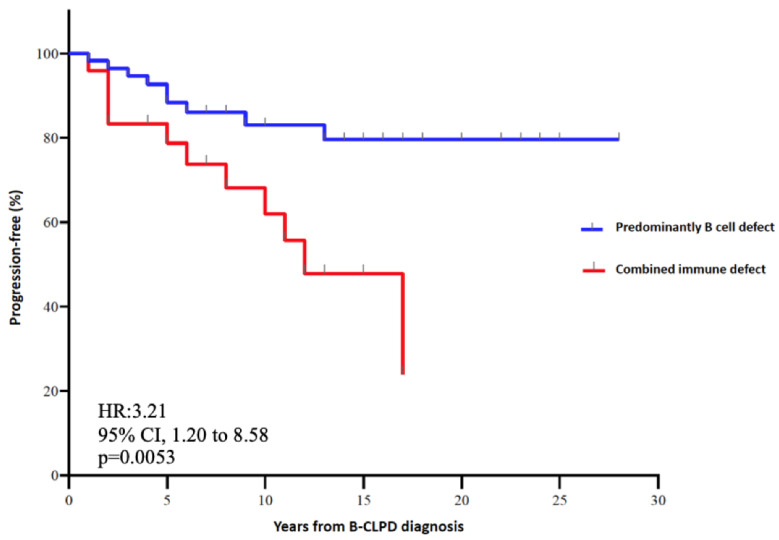
Kaplan–Meier plot of progression-free and hazard ratio according to the immune defect phenotype.

Importantly and not previously reported, patients with combined immune defect showed a 3.67-fold higher risk for cancer progression (95% CI, 1.38–9.72) than predominantly antibody defect (*p* = 0.014). By Kaplan–Meier estimates, patients with predominantly antibody defect had 80% of progression-free time compared with 20% in patients with combined immune defect phenotype. Combined immune defect showed earlier progression of hematologic cancer with a hazard ratio of 3.21 (95%CI, 1.20 to 8.58) than predominantly antibody defect (*p* = 0.005) (Figure 2). However, there were no significant differences in mortality rates between both categories (21% with combined immune defect vs. 15% with predominantly antibody defect (*p* = 0.5). Patients that died in the category of combined immune defect consisted of four patients with a decrease in complement and two patients with CD4^+^ T lymphocytopenia (<100 cells/µL), suggesting that accumulation of immune defect might impact on the clinical prognosis of B-CLPD patients. 

To assess whether the clinical stages of the disease or the oncologic treatment received could be a confounding factor, we stratified patients as follows: CLL, FL, MGUS, DLBCL; age matched (less than or equal to 65 years old, or greater than 65 years old), and as treated or untreated. We did not observe statistically significant differences among groups by Kaplan–Meier analysis. 

The main haematological diagnosis in patients with CD4^+^ T lymphocytopenia corresponded to NHL (75%) at different stages of the disease. We also stratified patients according to age and CD4^+^ T lymphocytopenia with no significant difference observed (*p* = 0.221). Regarding patients with low absolute counts of NKs cells, a heterogeneous distribution was observed (44% NHL, 33% CLL, and 23% in the paraprotein group). Decrease in C4 was prevalent in the MGUS (33%), CLL (22%) and MM (16%) patients’ groups. Lastly, the group with the greatest prevalence of persistent neutropenia were individuals with NHL diagnosis. 

## 6. Associated Conditions

Chronic or recurrent infections were found in all cases, the most common being pneumonia and bronchitis (Table 2). Upper respiratory tract infections were the most common infections (73% of patients). Lower respiratory tract infections as recurrent bronchitis occurred in 11%, pneumonia in 40%, and recurrent pneumonia (greater than 3 episodes during the observation period) in 30% (*n* = 10) of patients. During the follow-up period, 23% (*n* = 19) of patients associated with bronchiectasis. Patients with a previous history of pneumonia were at increased risk of developing bronchiectasis (*p* = 0.009). Thirteen (16%) patients were associated with at least one episode of severe infection (SI) in the year prior to the immunological evaluation. VZV infection occurred in 24% (*n* = 20) of the subjects and 20% (*n* = 4) had VZV recurrence. One patient had lung lymphangiomatosis and was proposed for lung transplantation, with suspected underlying PID. Unusual or opportunistic infections were also found, including both viral and fungal pathogens. Opportunistic infections were found in 18 patients (26%), all of them in patients with altered anti-TV response. One patient presented with severe CMV viral infection and human papilloma (VPH) reactivation associated with low NK cells. *Pneumocystis jirovecii* infection was developed in one patient with low CD4^+^ and CD8^+^ T lymphocytes counts. Campylobacter infection was reported in three patients, two of them with low NK cell counts. Aspergillus infection was diagnosed in four patients, with immune defect distribution as follows: predominantly antibody defect (*n* = 2); B^−^T^−^ (*n* = 1), B^−^C^−^ (*n* = 1). Five patients had a history of pulmonary TB infection, four of them with combined immune phenotype. Four patients had oropharyngeal candidiasis, all with predominantly antibody defect. Twenty-one patients (25%) were on active antibiotic prophylaxis due either to CD4^+^ T lymphocytopenia (<400 cells/µL) or to bronchiectasis with bacterial colonization.

### 6.1. Autoimmune Disease and Immune Dysregulation

Autoimmune conditions affected 10% (*n* = 8) of patients, with equal sex distribution. The most common autoimmune condition was AIHA (*n* = 3) followed by ITP (*n* = 2). The remaining three patients had psoriatic arthritis, SLE, primary biliary cholangitis (PBC) and Sjogren’s disease, treated with corticosteroids and immunomodulatory drugs—IVIg and rituximab were given for AIHA and ITP— One patient with PBC was treated with ursodeoxycholic acid. 

### 6.2. Gastrointestinal Involvement

Thirty-seven (44%) patients were screened for celiac disease (CD) due to malabsorption syndrome. None of them presented anti-tissue transglutaminase IgA nor anti-deamidated gliadin peptide IgG antibodies. However, 12 out of 37 (32%) which underwent genetic testing were associated with human leukocyte antigen (HLA)-DQ2 or DQ8 haplotype, a similar prevalence to the general population. Seven out of 12 (58%) presented with mild to severe malabsorptive symptoms, suggesting a seronegative celiac disease. In one of them (pt #18), endoscopy revealed a sprue-like illness with histopathologic analysis showing subtotal villous atrophy (Marsh 3b) without the typical CD immunophenotype of intraepithelial cells and non-responsive to wheat withdrawal. PID was suspected in this patient and whole exome sequencing (WES) analysis was requested. Specific causal infection by *Campylobacter jejuni* was documented in one patient. Thirty-two of 83 patients were tested for *Helicobacter Pylori* (HP) IgG antibodies prior to IgRT, 47% (*n* = 15) were positive and 33% (*n* = 5) were confirmed by endoscopy; only one patient was associated to MALT lymphoma with three lymphoma relapses. 

### 6.3. Second Primary Neoplasia

Nine patients (11%) were associated with second primary neoplasia (SPN), with a mean age since haematological malignancy diagnosis of 5.6 years, four patients were in the paraprotein group (MGUS, MM, Waldenström disease). The most common SPN were thyroid and breast cancer (Table 3). On the other hand, five patients had developed a primary neoplasia years before B-CLPD diagnosis, with a mean time lapse of 8.8 years (Table 4). Only one patient had a gastrointestinal neoplasia diagnosis before the haematological diagnosis associated to refractory *H. Pylori* infection, and genetic CD susceptibility. 

### 6.4. Suspicion of Underlying Primary Immunodeficiency Disease 

Twelve out of 83 patients (14%) were highly suspicious of an underlying PID, due to a history of infections since childhood, autoimmune and granulomatous complications, or PID-compatible biological data. Of interest, in one patient (#54) the lymphoma was the clinical onset of CVID, and whole exome sequencing (WES) analysis revealed heterozygous mutations in TACI and LRBA genes and an homozygous mutation in PLCG2 (somatic mutation described in cancer) with ibrutinib resistance and HL recurrence [34]. Another patient revealed heterozygous mutations in PLCG2. Functional characterization of the pathway of the affected gene is ongoing. PID genes observed were likely pathogenic, as we considered only variants with very low allelic frequency (prevalence below 0.005% in the general population). To see technical information about the WES methods and variant analysis please refer to Supplementary Methods. Interestingly, immunological data at diagnosis showed significantly reduced serum free κ and λ chain levels in 9 of 12 patients with PID suspicion (Table 5), suggesting CVID-like features [35]. 

Six suspected PID patients showed very low sFLC (0.76 ± 0.88 and 0.83 ± 0.89) in contrast to 30 NHL patients previously treated with rituximab, in which normalization of both kappa and lambda light chain levels were observed (12.3 ± 8.0 and 12.8 ± 9.0, respectively), suggesting that low levels of sFLCs might be a useful complementary biomarker for PID diagnosis despite cancer treatment.

## 7. Management and Therapeutic Strategies

### 7.1. Immunoglobulin Replacement Therapy 

At first documentation, 12 patients (22%) had received at least one dose of IVIg (30 g/dL) 2 years before immunological referral. After completing immunological evaluation, 42 patients (78%) were initiated on IgRT. A total of 54 patients were on active IgRT treatment, 60% (*n* = 50) on IVIg (dosing interval 3–5 weeks) and 5% (*n* = 4) were switched to SCIg (dosing interval 2–4 weeks) due to persistence of infections or due to patient’s preferences, and PID was suspected in two patients with persistent infections despite IgRT. Clinical improvement was observed 6 months after SCIg initiation, with a reduction in global infections from 4.0 (3.5–2.5) to 2.0 (1.5–0.5) (*p* = 0.18). As expected, the rate of bacterial infections in patients under IgRT substantially decreased compared with the previous 12 months (*p* < 0.0001). The average Ig dose was 300 mg/kg every 4 weeks for IVIg. Follow-up dosages remained stable and only four patients had dose changes. Overall, eight patients (9%) benefited from transitory therapy interruptions (“treatment holidays”) during follow-up. Typhim booster vaccination was performed to evaluate humoral reconstitution (Table 6). Only one patient was able to mount an adequate antibody response associated with clinical improvement, IgRT was discontinued, and the patient is closely followed.

### 7.2. Trained Immunity-Based Vaccines 

Thirty-one of 83 (37%) patients received mucosal sublingual vaccine for recurrent upper respiratory or urinary tract infections. In 23 patients, TIbV was used as a first-line prophylaxis in addition to antibiotic implementation during the period of immunological assessment. In five additional patients, TIbV was initiated concomitantly with IgRT treatment and in three patients as adjuvant therapy to IgRT due to the persistence of upper respiratory tract infections. Overall, 74% of patients showed a clear improvement on infection episodes. Remarkably, patients with a history of previous herpes infections presented a significant reduction from 3.33 (2.0–5.0) to 0.5 (0–1.25) (*p* < 0.001). Other significant results were the decrease in the number GP visits from 2.9 (2.0–3.5) to 0.7 (0–1.0) and in hospital admissions (*p* < 0.001). 

## 8. Prognostic

### Survival, Progression, and Mortality 

The median follow-up time of our cohort was 3.8 years. To date, fourteen (17%) patients died (10 males and 4 females). The mean age at the time of death was similar for both groups (80 years) (*p* = 0.06). The major cause of death was cancer progression and infectious complications. 

## 9. Discussion 

**Figure 3:**
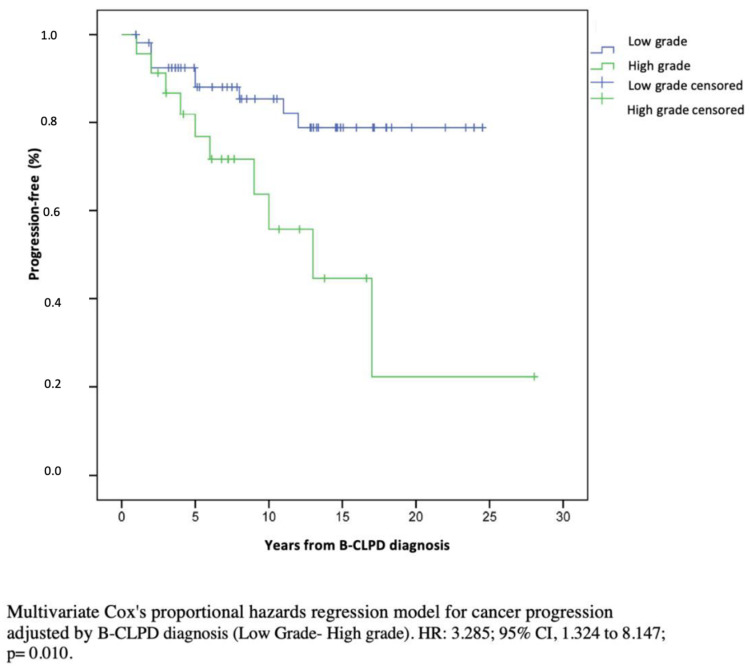
Multivariate Cox’s proportional hazards regression model for cancer progression adjusted by clinical stage (low grade/high grade).

The expression of SID in haematological cancer patients has been poorly characterized, especially due to the clinical heterogeneity of the different B-CLPD conditions. In this observational, single center and real-life cohort study of SID to B-CLPD patients, categorization of SID into predominantly antibody defect and combined immune defect was clinically significant both in terms of infectious and cancer progression risk. Indeed, patients with combined immune defect showed a 3.69-fold higher risk for severe infection and a 3.21-fold higher risk for cancer progression than those with predominantly antibody defect. To our knowledge, this is the first report jointly addressing the immunological phenotype as a predictive factor for the progression of haematological malignancy beyond infectious risk. By Kaplan–Meier estimates, 80% of patients with combined immune defect progressed vs. only 20% with predominantly antibody defect. Cancer treatment and immunosenescence are major important drivers in the degree of SID. B-CLPD are age-related diseases. The influence of age on immunity of tumors is not well known: on the one hand, advanced age impairs cell capability to eliminate cancer cells (immunosenescence); while on the other hand, advanced age is associated with chronic inflammation, favoring tumorigenesis and cancer progression [36]. Despite the nosological heterogeneity of B-CLPD, our results show that categorization of patients according to age (less than 65 or equal or greater than 65 years old), CD4^+^ T cell defect, and oncologic treatment did not show any statistically significant differences by Kaplan–Meier analysis. Differences in cancer progression and severe infections were, rather, associated with the degree of immunosuppression and stage of malignant disease. The mechanisms underlying cancer progression are complex and not clearly elucidated. Potential factors involved in our findings may relate to defective immunosurveillance favoring immune escape and tumor growth and progression. Combined immune defect was associated with severe infections and progression and/or recurrence after an independent analysis according to low grade, high grade and plasma cell dyscrasias classification (Figure 3).

The clinical relevance of infectious risk associated with B-CLPD was described more than 30 years ago [4], although progress in the field has been limited and frequently extrapolated from experience in PID patients [37]. PID and SID are quite different conditions that should be properly and differentially evaluated. For instance, SID may preserve normal secondary specific antibody responses to pneumococcus and tetanus toxoid while lacking primary antibody responses, which may be evaluated through immunization with neoantigens, such as the polysaccharide of *S. Typhi* [12,13,14,38,39], showing different cut-off levels for functional antibody responses [14]. In our B-CLPD cohort, predominantly antibody defect represented 71% of SID patients as the only detectable defect. Our results indicate that, although B cell defect is the earliest and most common defect in B-CLPD patients, alterations of other immune cell types might impact the outcome [40,41]. In our cohort, 11% patients had an associated decrease in C4 concentrations, of which 44% died, representing almost a third of total deaths. Regarding patients with a decrease in C4, given that functional studies were not performed, the functional significance is not known. None had clinical or other analytical data suggestive of active autoimmune disease at the time of the immunological evaluation. Double edge roles in complement and tumor progression have been described, as a defect in C4 complement may slow tumor growth, while C4 complement activation has pro-inflammatory and, thus, pro-tumoral properties [42]. However, considered just as a possible biomarker, a decrease in C3 or C4 is an interesting finding. Testing the functional activity of the complement system would, in future studies, deepen knowledge of the pathophysiology. 

In our cohort, around 14% of SID were “suspected PID patients”. Among SID patients, there is a subgroup of yet unknown magnitude with undiagnosed PID. Viral or bacterial infections are more frequent in the setting of PID, but intrinsic complex oncogenic mechanisms may play a major pathogenic role, for instance: stem cell developmental defects; lymphocyte differentiation and apoptosis defects; co-stimulation, cytoskeleton and signalling defects; cell cycle and cytokinesis defects; DNA repair, chromosome stability and telomere maintenance defects - [43]. The proportion will presumably increase as we dissect the genetic convergence of PID and haematological cancer [44]. Hence, PID screening at cancer diagnosis is justified [45]. An increased susceptibility to haematological malignancies varies according to PID category, in which B-CLPD is the most frequent, and maybe the initial, manifestation [43]. A bias in comparing SID and “suspected PID”, therefore, existed, but we could not avoid it, as PID patients were not confirmed given the complexity of genetic analysis and their phenotypic expression. PID patients with subsequent B-CLPD may have greater risk of infections, toxicities to antitumor regimens, cancer recurrence and SPN and there is an ongoing effort to adjust cancer protocols for PID patients [46]. The size of “suspected PID” vs. “SID” did not permit us to draw conclusions in terms of infectious risks, autoimmune complications and second primary neoplasia.

Interestingly, 75% of the “suspected PID patients” showed very low or undetectable sFLCs, in line with previous work in CVID. Although sFLCs are a good real-time indicator of tumor burden, an unresolved question is whether part of this reduction could be related to cancer treatment, especially in patients with a possible underlying PID. Recent studies indicate an earlier reduction in sFLC after treatment compared with other biomarkers [47]. On the other hand, fluctuating levels of sFLC have been associated with temporary tumor inhibition and may be a marker of tumor resistance. Our results add to the potential value of sFLC as biomarker for underlying CVID diagnosis despite cancer treatment.

The delay from haematological malignancy diagnosis to immunological referral and evaluation was 7 years, without difference between patients with predominantly antibody defect or combined immune defect. It would be interesting to ascertain if early immunological evaluation and prophylactic intervention may impact survival. 

Identification of SPN in our cohort was an additional important observation. The median period to develop a SPN after B-CLPD diagnosis was 2.5 years for CLL, 3 years for NHL and 5 years for MGUS. A shorter time interval for SPN in B-CLPD than conversely was observed, maybe reflecting the lack of immune surveillance due to B-CLPD or therapy. Although the exact mechanisms involved in the development of SPN are not well characterized, several factors—such as chemoimmunotherapy protocols, genetic predisposition, cumulative gene lesions, dysregulation of immune surveillance, chronic inflammation as well as better diagnostic tools, etc.—have been reported [48]. Published data indicate that for patients with CLL, it occurs within the first 5 months after diagnosis, and between 5 to 10 years in patients with NHL, HL, MM and MGUS [49,50,51,52]. Enhanced screening practices depending on the patient’s age and risk factors can be of vital importance. 

Another important aspect in the evaluation of SID patients is the screening for enteropathy. In our cohort, seven patients with long-term malabsorptive symptoms gave rise to suspicion of a concomitant undiagnosed disease, such as sprue-like illness, *Campylobacter jejuni* infection. Therefore, it is important to consider the best diagnosis screening and interdisciplinary management, allowing prompt diagnosis and prevention of long-term complications.

Even through the changing spectrum of infections in patients on current therapeutic protocols, encapsulated bacteria and herpesviruses represented the most prevalent infections in our cohort, corresponding to 88% and 24%, respectively, similar to that described in previous publications [53,54]. Hence, prevention of infections is an important strategy to improve quality of life and prognosis in cancer patients. The use of antibiotics during immunological assessment has been previously agreed [23,24,30,31] and remains the mainstay of management in patients with recurrent infections, showing improved survival in patients with prolonged neutropenia [55]. However, prolonged prophylactic antibiotics might have deleterious effects. Fifty-four patients (65%) of our cohort received IgRT prophylaxis and as expected, the rate of bacterial infections in patients decreased significantly compared with the previous 12 months. A third of our patients were given (TIbV) as an adjuvant immune intervention on SID management [27]. TIbV might support the long-lasting epigenetic changes in innate immune cells, enabling quicker clearance of microbial infections. TIbV cross-protected against both bacterial and viral (herpes simplex, VVZ) infections, compatible with previous reports [56,57,58]. 

The heterogeneity of our cohort is one of the main limitations of our study and conclusions cannot be drawn for the small groups of patients (e.g., Waldenström, MM and HL, LLA). Nevertheless, our study has several strengths, including the real-life scenario weight of different immunological parameters, easily available in most standard clinical care settings. Additionally, we point to the need to look for other associated conditions, such as SPB, autoimmune disease, gastrointestinal involvement.

## 10. Conclusions

Our results point to the added value of evaluating innate and adaptive immune defects for better predicting severity in patients with B-CLPD, independently of the disease. We believe that understanding the process of tumour spread through the prism of the immune system may provide a more integrative insight into clonal growth and immunodeficiency affecting the individual patient. Future studies are warranted to confirm the clinical relevance of immunological categorization. 

## Figures and Tables

**Table 1:** Immunological characteristics of the main cohort groups.

Variable	NHL (No. 41)	CLL (No. 18)	MGUS (No. 12)
IgG (mg/dL)	516 ± 319 435 (471)	480 ± 335 338 (481)	1423 ± 797 1200 (1144)
IgA (mg/dL)	59 ± 70 19 (87)	62 ± 122 17 (33)	133 ± 97 94 (207)
IgM (mg/dL)	67 ± 124 26 (60)	23 ± 25 14 (14)	233 ± 378 91 (131)
IgG1 subclass (mg/dL)	311 ± 198 285 (328)	235 ± 142 196 (192)	914 ± 588 905 (729)
IgG2 subclass (mg/dL)	170 ± 128 120 (206)	201 ± 178 118 (301)	284 ± 184 264 (199)
C3 (mg/dL)	127 ± 29 129 (38)	129 ± 37 131(63)	114 ± 28 118 (40)
C4 (mg/dL)	29 ± 7 28 (9)	25 ± 11 25 (14)	20 ± 10 18 (11)
CD4^+^T-lymphocytes/mm^3^	521 ± 324 494 (419)	1177 ± 931 1082 (927)	852 ± 434 774 (739)
CD8^+^T-lymphocytes/mm^3^	612 ± 612 515 (362)	1870 ± 3051 985 (1143)	534 ± 257 521 (495)
CD19 B-lymphocytes/mm^3^	60 ± 121 103 (160)	17,328 ± 3337 4722 (11,402)	173 ± 53 143 (73)
NK cells/mm^3^	223 ± 193 139 (256)	697 ± 651 591 (820)	256 ± 132 229 (225)
Neutrophils ×10^3^/uL)	3572 ± 1886 3450 (2350)	3508 ± 1425 3640 (2492)	3650 ± 1884 3850 (1900)

Data are presented as mean ± standard deviation; median (interquantilic range, IQR). Reference values: serum immunoglobulins (mg/dL) IgG: 767–1590; IgA: 61–356; IgM 37–286. Immunoglobulin subclasses (mg/dL) IgG1: 341–894; IgG2: 171–632. Complement system: C3: 70–140 mg/dL, C4: 15–30 mg/dL. Reference values for CD4^+^ T-lymphocytes: 51–66%; 464–1721; CD8^+^ T-lymphocytes: 28–36%; 178–853; CD19 B lymphocytes: 7–13%; 92–515; NK cells CD3^−^CD56^+^ CD16^+^: 8–19%; 82–594.

**Table 2:** Past medical history of B-CLPD patients.

	No. of Patients *n* = 83	%
Recurrent bronchitis, sinusitis, otitis	73	88
Pneumonia	33	40
Sepsis (*Pseudomona* sp., *pneumococcus*, *H. influenzae*, *S. agalactiae*, CMV)	22	27
History of herpes zoster	20	24
Recurrent urinary tract infections	12	14
Recurrent oral herpes	7	8
Pulmonary TB	5	6
Oropharyngeal candidiasis	4	5
Viral hepatitis	4	5
Aspergillosis (pneumonia, brain abscess)	4	5
Campylobacter enteritis	3	4
Cellulitis	3	4
Cytomegalovirus pneumoniae	2	3
Human papillomavirus reactivation	2	3
Meningitis	1	1
Cryptogenic organizing pneumonia	1	1
Recurrent parotitis	1	1
*Pneumocystis jirovecii* infection	1	1
Osteomyelitis	1	1
Pyoderma gangrenosum	1	1

**Table 3:** Time interval between diagnosis of B-CLPD and a second primary neoplasia diagnosis in our cohort of B-CLPD patients.

Patient	B-CLPD	Immune Defect	Time Interval (Years)	SPN
#4	CLL	Predominantly Ab defect	1 (2013–2014)	Lung adenocarcinoma
#12	CLL	Combined immune defect	4 (2005–2009)	Thyroid papillary carcinoma
#18	NHL (FL)	Predominantly Ab defect	3 (2003–2006–2008)	Peripheral nerve sheath tumor, Thyroid cancer and malignant nasal Ca
#28	NHL (FL)	Predominantly Ab defect	4 (2013–2017)	Colon adenocarcinoma
#48	NHL	Predominantly Ab defect	22 (1992–2014)	Prostate adenocarcinoma
#60	MGUS	Predominantly Ab defect	6 (2006–2012)	Breast cancer (infiltrating ductal carcinoma)
#61	MM (IgA kappa)	Predominantly Ab defect	7 (2013–2020)	Pancreatic intraductal papillary mucinous neoplasm
#62	MGUS (IgA Lambda)	Predominantly Ab defect	3 (2016–2019)	Breast cancer (infiltrating ductal carcinoma)
#68	MGUS (IgA Lambda)	Predominantly Ab defect	1 (2016–2017)	Endometrial cancer

SPN: Second primary neoplasia.

**Table 4:** Time interval between a primary neoplasia diagnosis and B-CLPD diagnosis.

Patient	Primary Neoplasia	Time Interval Years	Immune Defect	B-CLPD Diagnosis
#5	Basal cell carcinoma	5 (1998–2003)	Predominantly Ab defect	CLL
#24	Breast cancer	14 (1994–2008)	Combined immune defect	NHL (FL)
#25	GIST	2 (2009–2011)	Combined immune defect	NHL (FL)
#43	Prostate adenocarcinoma	12 (2002–2014)	Predominantly Ab defect	NHL (DLBCL)
#59	Breast cancer (infiltrating ductal carcinoma)	11 (2005–2016)	Combined immune defect	MM

GIST: Gastrointestinal stromal tumor.

**Table 5:** Patients with suspected primary immunodeficiency.

Patient	B-CLPD Diagnosis	Age at Diagnosis (Years)	Date Last Chemotherapy	Serum Free Kappa (mg/L)	Serum Free Lambda (mg/L)	Past Medical History Previous B-CLPD Diagnosis	GI Involvement
#7	CLL	50	2012	2.0	1.5	RRTI; Salmonella GI.	No *Salmonella Typhi* gastroenteritis
#10	CLL	38	2020	12.9	5.8	RRTI	Genetic susceptibility to CD
#12	CLL	48	2017	12.3	12.3	RRTI	Persistent *H pylori* infection
#15	CLL	54	2007	0.1	0.3	RRTI	*Campylobacter jejuni* infection
#18	NHL (FL)	67	2008	0.0	0.0	Severe infection	Celiac disease (MARSH III)
#23	NHL (FL)	50	1996	0.0	0.0	Recurrent pneumonia	-
#32	NHL (FL)	45	2017	0.3	0.3	RRTI	-
#34	NHL (FL)	34	2009	0.3	1.1	RRTI, zoster infection.	-
#42	NHL (DLBCL)	59	2014	2.3	1.7	Pneumonia	Persistent *H. pylori* infection
#51	NHL (Burkit)	7	2017	2.1	1.5	Lymphoma recurrence	Persisten *H. pylori* infection
#54	HL	13	-	2.1	1.7	RRTI, lymphoma recurrence	Genetic susceptibility to CD, elevated liver enzymes, *H. pylori* infection
#63	MGUS	41	-	19.0	25.0	Lung lymphangiomatosis	Genetic susceptibility to CD

RRTI: recurrent respiratory tract infection; CD: celiac disease.

**Table 6:** Evaluation on humoral reconstitution after 12 months of IgRT by measuring Typhim Vi booster response.

Patient	B-CLPD Diagnosis	Baseline	Post-Vaccination	Typhim Vi Booster
#3	CLL	7.4	7.4	12.4
#4	CLL	7.4	7.4	8.8
#7	CLL	7.4	7.4	15.4
#8	CLL	7.4	10.3	32.1
#10	CLL+HL	7.4	7.4	12.4
#19	NHL	7.4	7.4	9.2
#25	NHL	7.4	7.4	7.4
#50	NHL (MALT)	7.4	7.4	7.4

## Supplementary Materials

The following supporting information can be downloaded at: https://www.mdpi.com/article/10.3390/biomedicines10082020/s1, Table S1: B-cell lymphoproliferative disorders study groups according to hematological disease; Table S2: Defective specific antibody response of the main cohort groups.

## Abbreviations

B cell lymphoproliferative diseases (B-CLPD), secondary immunodeficiency (SID), immunoglobulin replacement therapy (IgRT), antibody (Ab), *Salmonella typhi* vaccine (TV), dendritic cells (DCs), natural killer (NK) cells, primary immunodeficiency (PID), trained immunity-based vaccines (TIbV), recurrent upper respiratory tract infection (URTI), recurrent lower respiratory tract infection (LRTI), recurrent urinary tract infection (RUTI), chronic obstructive pulmonary disease (COPD), varicella-zoster (VZV), 23-valent pneumococcal polysaccharide (PPV) vaccine, tetanus-toxoid (TT) vaccine, non-Hodgkin lymphoma (NHL), chronic lymphocytic leukaemia (CLL), monoclonal gammopathy of undetermined significance (MGUS), multiple myeloma (MM), Hodgkin lymphoma (HL), Waldeström disease (WD), lymphoblastic acute leukaemia (LAL), follicular lymphoma (FL), LPS responsive beige-like anchor protein (LRBA), transmembrane activator and CAML interactor (TACI), phospholipase C gamma 2 (PLCG2).
