# Supplementary material for: Combined Immune Defect in B-Cell Lymphoproliferative Disorders Is Associated with Severe Infection and Cancer Progression
Source: Biomedicines. 2022 Aug 19;10(8):2020. doi: 10.3390/biomedicines10082020 (PMC9406016; doi:10.3390/biomedicines10082020)
Supplement: Supplementary file 1 [file biomedicines-10-02020-s001.zip › biomedicines-1780603-supplementary.pdf]

Supplementary material

Supplementary Table S1. B-cell lymphoproliferative disorders studied groups according to hematological disease.

**Hematological disease**	**No. of patients**
NHL	41
CLL	18
MGUS	12
MM	6
HL	3
Waldeström disease	2
Acute lymphoblastic leukemia	1

Abbreviations: non-Hodgkin lymphoma (NHL), chronic lymphocytic leukemia (CLL), monoclonal gammopathy of undetermined significance (MGUS), multiple myeloma (MM), Hodgkin lymphoma (HL), Waldeström disease (WD) and lymphoblastic acute leukemia (LAL).

Supplementary. Table S2. Defective specific antibody response of the main cohort groups.

	**Anti-TT**	**Anti-PCP**	**Anti-S. typhi**
NHL	89%	97%	97%
CLL	100%	83%	100%
MGUS	66%	92%	75%

Anti-TT: tetanus-toxoid; PCP: pneumococcal, S. typhi: Salmonella typhi. Adapted from Ochoa-Grullón et al. Clinical Immunology 2020.
